# YRDC Mediates the Resistance of Lenvatinib in Hepatocarcinoma Cells via Modulating the Translation of KRAS

**DOI:** 10.3389/fphar.2021.744578

**Published:** 2021-10-01

**Authors:** Jun Guo, Peng Zhu, Zhi Ye, Mengke Wang, Haijun Yang, Shiqiong Huang, Yan Shu, Wei Zhang, Honghao Zhou, Qing Li

**Affiliations:** ^1^ Department of Clinical Pharmacology, Xiangya Hospital, Central South University, Changsha, China; ^2^ Institute of Clinical Pharmacology, Hunan Key Laboratory of Pharmacogenetics, Central South University, Changsha, China; ^3^ Engineering Research Center of Applied Technology of Pharmacogenomics, Ministry of Education, Changsha, China; ^4^ National Clinical Research Center for Geriatric Disorders, Changsha, China; ^5^ Department of Anesthesiology, Xiangya Hospital, Central South University, Changsha, China; ^6^ Department of Pharmaceutical Sciences, School of Pharmacy, University of Maryland at Baltimore, Baltimore, MD, United States

**Keywords:** YRDC, Lenvatinib, HCC, t^6^A modification, KRAS

## Abstract

Lenvatinib is the latest and promising agent that has demonstrated a significant improvement of progression-free survival in advanced hepatocellular carcinoma (HCC). However, resistance emerges soon after initial treatment, limiting the clinical benefits of lenvatinib. Therefore, understanding the mechanism of resistance is necessary for improving lenvatinib efficacy. YRDC promotes the proliferation of hepatocarcinoma cells via regulating the activity of the RAS/RAF/MEK/ERK pathway, which was the primary pathway of the anticancer effect of lenvatinib. The purpose of this study is to investigate whether YRDC modulates the sensitivity of lenvatinib in hepatocarcinoma cells. Using the CCK-8 cell viability assay, wound-healing assay and clone formation assay in cell models, and xenograft assay in null mouse, we demonstrated that Huh7 cells with YRDC knockdown showed decreased susceptibility to lenvatinib than their control cells. Furthermore, we found that lenvatinib inhibited the expression of YRDC in a time-dependent manner. This effect may aggravate resistance to lenvatinib in hepatocarcinoma cells and may be an underlying cause of resistance, which emerges soon after lenvatinib initial treatment. To investigate how YRDC modulates the sensitivity of lenvatinib, we assessed the effect of tRNA with different t^6^A levels on the translation of the KRAS gene by *in vitro* rabbit reticulocyte translation system and measured the expression levels of the KRAS gene by western blot together with qPCR. We found that YRDC regulates the protein translation of KRAS in cell models, and the tRNA with low t^6^A modification level reduces the translation of the KRAS in the *in vitro* translation system. These results suggested that YRDC mediates the resistance of lenvatinib in hepatocarcinoma cells via modulating the translation of the KRAS. In this study, YRDC was confirmed to be a potential novel predictive biomarker of lenvatinib sensitivity in HCC.

## Introduction

Hepatocellular carcinoma (HCC) is the fourth leading cause of cancer death worldwide and has a higher growth incidence (Bray et al., 2018). The estimated incidence of new cases is approximately 500,000–1,000,000 annually, causing 600,000 mortalities globally per year. More than half of all cases are from China (Zheng et al., 2018). About 30–40% of patients are diagnosed at early stages. Potentially curative treatment of these patients, such as locoregional therapies and surgical therapies, can achieve 5-year survival rates up to 60–70% (European Association for the Study of the Liver, 2018; Llovet et al., 2018; Nault et al., 2018). However, there are few effective therapies for patients with advanced stage or with tumor progression after locoregional, leading to an extremely poor prognosis. Traditional chemotherapy drugs have little effect on survival in patients with advanced HCC (European Association for the Study of the Liver, 2018).

Sorafenib, an oral multikinase inhibitor (MKI), was approved as a unique target drug for advanced HCC in 2007 and has been used as a first-line systemic treatment for patients with unresectable HCC (uHCC) (Llovet et al., 2008). However, after clinical application of sorafenib for over a decade, the long-term survival for patients with uHCC is still suboptimal (Llovet et al., 2008; El-Serag, 2011; Zhu, 2012). In the last few years, several MKIs have been developed and clinically tested for the treatment of uHCC, but none of them has exhibited superior efficacy to sorafenib (European Association for the Study of the Liver, 2018; Heimbach et al., 2018; Vogel et al., 2018; Al-Salama et al., 2019).

Lenvatinib is an emerging MKI that targets the vascular endothelial growth factor receptor (VEGFR) one to three, fibroblast growth factor receptors (FGFR) one to four, platelet-derived growth factor receptor (PDGFR) α, and proto-oncogenes RET and KIT (Tohyama et al., 2014; Yamamoto et al., 2014). Firstly, it was approved for the treatment of advanced renal cell carcinoma (RCC) and differentiated thyroid cancer (DTC) (Schlumberger et al., 2015). In June 2017, the American Society of Clinical Oncology reported that lenvatinib was the first drug to show noninferiority to sorafenib in terms of overall survival in the phase III REFLECT trial in patients with uHCC. In addition, lenvatinib demonstrated a significantly higher objective response rate, longer progression-free survival, and time to progression compared to sorafenib (Al-Salama et al., 2019). Based on all these promising results from the REFLECT trial, lenvatinib is promptly approved for the first-line treatment of patients with uHCC in the USA, the EU, Japan, and China in 2018 total population (Inc, 2018). In fact, despite therapy, most patients with HCC or DTC develop resistance to lenvatinib after a period of time (Capozzi et al., 2019; Bangaru et al., 2020; Fu et al., 2020). For these reasons, it is urgent to identify a biomarker to predict the response or resistance to lenvatinib.

YRDC belongs to the universal family with the sua5-yciO-yrdC domain (PF01300, Swiss-Prot/TrEMBL database) and is highly conserved from *E. coli* to *Homo sapiens* (Jia et al., 2002). YrdC/Sua5 family is involved in the N6-threonylcarbamoyladenosine (t^6^A) biosynthesis of tRNA that recognize ANN codons in *E. coli* and yeast. The t^6^A modification is one of the 15 universally conserved modification located at position in tRNA decoding ANN codons (Cantara et al., 2011). Structural studies showed that t^6^A modification could strengthen the codon–anticodon interaction and promote translational fidelity by the ribosomes (Weissenbach and Grosjean, 1981; Grosjean et al., 1995; Yarian et al., 2000). These findings implicate that YrdC/Sua5 plays a critical role in protein translation at the level of codon recognition.

Our previous study reported that the expression of YRDC was dysregulated in hepatocellular carcinoma tissues. The results of *in vitro* and *in vivo* experiments showed that the expression of YRDC was positively associated with the proliferation and metastasis of HCC cells, and the mechanism exploration demonstrated that YRDC probably activates the MEK/ERK signaling pathway (Huang et al., 2019). Lenvatinib inhibits RTK receptors and subsequently blocks its downstream RAS/RAF/MEK/ERK signal transduction pathway, eventually impeding tumor cells proliferation and tumor angiogenesis (Leonetti et al., 2017; Cabanillas and Takahashi, 2018). These information suggested that HCC cells or tumor with high expression of YRDC, containing higher basal activity of the MEK/ERK, might be more sensitive, or responsive, to lenvatinib, and that those with low expression of YRDC might be resistant to lenvatinib. However, whether YRDC expression contributes to lenvatinib resistance has not yet been reported. Therefore, we conducted this study to investigate the association of the efficacy of lenvatinib with the expression of YRDC and explore the mechanism detail behind this association.

## Materials and Methods

### Cell Culture and Establishment of Stable Cell Lines

Huh7 (TCHu182) and HepG2 (TCHu72) cell lines were purchased from Shanghai Institute of Biochemistry and Cell Biology, Chinese Academy of Sciences. The culture medium of these 2 cell lines was Dulbecco’s Modified Eagle’s Medium supplemented with 10% fetal bovine serum (FBS) (Gibco, Carlsbad, CA, United States). The cells were cultured in an incubator at 37°C and 5% CO_2_. Short hairpin (sh) RNAs were purchased from Shanghai GenePharma Co., Ltd. The sequences of shYRDC and shNC were used as follows: shYRDC: 5′- CAT​TCG​GAT​TCC​TGA​TCA​T -3′; and shNC: 5′- TTC​TCC​GAA​CGT​GTC​ACG​T -3′. Then, sh*YRDC* and shNC were cloned, respectively, into the BamHI and EcoRI sites of the pGLV3 lentiviral vectors (GenePharm). The YRDC gene was amplified with oligonucleotides to add the NotI and BamHI sites with the primers 5′- AGG​GTT​CCA​AGC​TTA​AGC​GGC​CGC​GCC​ACC​ATG​TCT​CCG​GCG​CGT​CGG​TGC​AGG​GGG​ATG -3′ and 5′- ATC​AGT​AGA​GAG​TGT​CGG​ATC​CTC​ACA​GGT​AGG​ACG​CAT​GTG​AGG​GGA​GCA​GTC​C -3′. The amplified products were cloned into the pGLV5 lentiviral vector (GenePharm). The empty pGLV5 lentiviral vector was used as a control. Huh7 or HepG2 cells were seeded at 2×10^4^ cells/well in 24-well plates, and 24 h after seeding were treated with a linear range of puromycin concentration. Lentivirus was diluted 10-fold with DMEM (supplemented with 10% FBS) for transduction. Cells were incubated with diluted lentivirus solution (Liu YB. et al., 2018). After 24 h, the culture medium was changed to a complete medium and selected with puromycin for 4 days. Until control, the Huh7 cells or HepG2 cells completely died, the YRDC stably knockout and overexpression cells were established. The optimal screening concentration of puromycin was 1 μg/ml. The lentivirus infected cells were observed under a fluorescence microscope (EVOS M5000, Invitrogen) and then collected for analysis by quantitative real-time PCR (qPCR) and western blot.

### Survival Assay

The Cell Counting Kit-8 (Bimake) assay was used to estimate the viability of the cells. Huh7 and HepG2 cells were seeded in 96-well plates (5,000 cells/well). Next, different concentrations of lenvatinib (Selleck) or BGJ (Selleck) were added into each well separately and incubated for 48 h. After that, the culture medium containing lenvatinib or BGJ was sucked out. The cells were washed with phosphate buffer solution (PBS). Then, the mixture of 10 μl CCK-8 solvent and 90 μl of fresh medium was added to each well and incubated for 60 min. Finally, the absorbance at 450 nm was measured using a microplate reader (EON, BioTek Instruments, Inc.). The cell survival rate was calculated. The experiment was repeated four times.

### Wound-Healing Assay

The cells were seeded in 6-well plates, and 2 ml of cell suspension with a density of 2×10^5^ cells/ml was added to each well. When the cell growth density was around 90%, a 100 μl pipette tip was used to gently scrape off the cells to create a scratch. PBS was added to wash the floating cells, and subsequently, the medium with lenvatinib was added into each well. Photos were taken with the Leica 3000 microscope, using a magnification of 100 times, at 48 and 72 h. ImageJ 1.8.0 (Bethesda, United States) was used for image processing. The experiment was repeated three times.

### Clone Formation Assay

The cells were seeded in 6-well plates at a density of 1,000 cells/per well. After the cells were stabilized, half of the wells were treated with 0.1 μM lenvatinib for 10 days. Lastly, the colonies were fixed with 4% paraformaldehyde (Beyotime, CHN) for 15 min and stained with crystal violet (Beyotime, CHN) overnight. Fifty cells are defined as a colony. The experiment was repeated three times.

### Quantitative Real-Time Polymerase Chain Reaction

Total RNA was isolated from the cells and tissues using RNAiso Plus (Takara) and reverse-transcribed using a PrimeScript™ RT reagent kit (Takara); the amount of RNA used in this experiment is 1,000 ng. The reverse-transcribed products were used as templates for qPCR with SYBR Green (Takara). Then, qPCR was performed using LightCycler^®^ 480 Instrument (Roche, Basel, Switzerland). Data were analyzed using the 2^-∆∆ct^ method, and the expression of GAPDH was used as normalization control. The primers (synthesized by Sangon Biotech, CHN) used in this study were listed as follows: YRDC, forward: 5′-GCA​AGC​GGA​CCC​TCA​AAC​AT-3′; reverse:5′-GCTCAACAAGGACCTAAACCCT-3′; GAPDH, forward: 5′-AGA​TCA​TCA​GCA​ATG​CCT​CCT​G-3′; reverse: 5′-TTG​GCA​TGG​ACT​GTG​GCA​TG-3′; KRAS, forward: 5′- GGG​GAG​GGC​TTT​CTT​TGT​GTA-3′; reverse: 5′- GTC​CTG​AGC​CTG​TTT​TGT​GTC-3′; FGFR2, forward: 5′-AGTCAAGTGGATGGCTCCAG-3′; and reverse: 5-ACAGTT-CGTTGGTGCAGTTG-3′. The experiment was repeated at least three times.

### Animal Experiments

All animal experiments were performed in accordance with the National Institutes of Health’s Guide for the Care and Use of Laboratory Animals and were approved by the Institutional Review Board of Central South University (Changsha, China). Forty female 5-week-old BALB/C nude mice (Beijing Vital River Laboratory Animal Technology Co., Ltd.) were fed under specific pathogen-free conditions at the Department of Laboratory Animals, Central South University. The mice were housed in a temperature-controlled environment of 23 ± 1°C with a 12 h light/dark cycle (lights on 08:00 AM) and given a standard mouse diet, with water provided *ad libitum*. Forty female 5-week-old BALB/C nude mice were randomly divided into four groups of 10 each. YRDC–KD, YRDC–OE, and their control Huh7 cells (NC–KD or NC–OE, 1 × 10^7^) were mixed with the Matrigel in a serum-free medium and then injected subcutaneously into the right lower quadrant of the nude mice. After 14 days, each group of mice was divided into two groups again. Lenvatinib (30 mg/kg) and solvent were intragastrically administered once a day. And, the tumor volume was measured every 2 days. All mice of YRDC–OE and its control were euthanized after 16 days; however, all mice of YRDC–KD and its control were euthanized after 26 days. Tumor nodules were removed and weighed. According to the formula: V = L × W^2^/2, where V is the volume (mm^3^), L is the maximum diameter (mm), and W is the shortest diameter (mm).

### High PerformanceLiquid Chromatography–Mass Spectrometry Analysis of t^6^A Modification

The isolated bulk tRNA was enzymatically hydrolyzed to nucleosides as described (Crain, 1990). In brief, we added 0.01 units of snake venom phosphodiesterase (Sigma), 10 units of nuclease P1 (Sigma), and 3 μl alkaline phosphatase (Sigma) in a 100 μl volume to digest approximately 100 μg of bulk tRNA. The digested extracts were directly injected in a HPLC–MS system for the separation and identification of the nucleosides as previously described (Pomerantz and McCloskey, 1990). HPLC–MS was performed with an ExionLC AD instrument (AB SCIEX, USA) coupled to a mass spectrometer (Triple Quad 6,500+, AB SCIEX, USA). A Discovery C_18_ (250 mm, 4.6 mm, 5 μm) reverse-phase column (Waters) equipped with an Ultrasphere ODS guard column (Beckman) was used for lipid separation. The nucleosides were eluted at a flow rate of 1 ml/min and a column temperature of 30 ℃ with a gradient consisting of 10 mM ammonium acetate (A) and 40% acetonitrile (B) as follows: 0–7 min 99% A, 7–30 min 95% A, 30–40 min 85% A, 40–50 min 95% A, and 50–60 min 99% A. The mass spectrometer records in the cation mode. Using multiple/selected response monitoring modes (MRM/SRM), the collision energy is found to be 10 eV. For adenosine, the mass shifts from m/z 268–m/z 136, and for t^6^A, the mass shifts from m/z 413.2–m/z 281. The experiment was repeated three times.

### *In vitro* Transcription

The cDNA of human KRAS was synthesized and inserted between EcoRI and SalI sites of pET-28a (+) by GeneChem. The constructed pET-28a (+)-KRAS plasmid was linearized by dissecting with XhoI. The KRAS mRNA was transcribed from 1 μg linearized plasmid DNA *in vitro* using the MEGAscript T7 kit (Ambion) in the presence of m7G (5′) ppp (5′) G (CAP) as described by the manufacturer. The KRAS mRNA was purified by phenol/chloroform extraction, ethanol precipitation, and quantified by UV spectroscopy.

### Chromatographic Depletion of Endogenous tRNAs

The specific depletion of endogenous tRNAs from commercial Flexi rabbit reticulocyte lysate (RRL) system (L4540, Promega) was achieved by ethanolamine–sepharose column chromatography according to the method of Jackson et al. (2001). All experimental steps were performed at 4 °C. 100 μl of 50% ethanolamine–sepharose slurry in buffer A (0.1 mM EDTA, 25 mM KCl, 1.1 mM MgCl_2_, 10 mM NaCl, 10 mM HEPES-KOH, and pH 7.2) with 50 mM KOAc and 0.25 mM Mg(OAc)_2_ was incubated with commercial RRL for 45 min at 4°C for the depletion of tRNA in commercial RRL. The desired supernatant (RRL depleted of endogenous tRNA) was collected by brief centrifugation at 1,500 *g* and was either used immediately for translation reactions or stored at −80 ℃.

### *In vitro* Translation

Cellular tRNAs were prepared from four kinds of Huh7 cells by the procedure of Dittmar et al. (2006). *In vitro* translation was performed using a RRL system (L4540, Promega). 1 μg of luciferase mRNA (L4561, Promega) or 2 μg of KRAS mRNA was added to RRL containing 70 mM KCl, 2 mM DTT, 10 μM minus leucine, 10 μM minus methionine, and 40 U/μl RNase ribonuclease inhibitor. For RRL that is depleted of endogenous tRNA, 2.5 μg of extra tRNA isolated from the four kinds of Huh7 cells was respectively added to the reaction mixture. A total volume of 50 μL from this reaction mixture was incubated for 60 min at 30 ℃. To avoid aminoacyl tRNAs producing background bands (∼25 kDa), we added 0.2 mg/ml RNase A to the reaction (after completion) and incubate for 5 min at 30 ℃. Luciferase assay was performed according to the protocol by using a luminometer (Sirius, Berthold) to quantify the luciferase produced in the *in vitro* translation system. The KRAS protein produced in the *in vitro* translation system was measured by the western blot. The experiment was repeated three times.

### Western Blot

The cells were collected from the cell flask into a 1.5 ml EP tube, and it was found that the number of cells is about 1 × 10^7^. Then, 1 ml RIPA lysis solution containing 10 μl protease inhibitor and 10 μL phenylmethanesulfonyl fluoride (P0013B, Beyotime) was added. The mixture was added to the cells and tissues under the conditions at 4 ℃ and fully dissolved for half an hour for protein extraction. The lysate was centrifuged at 8,000 g at 4°C for 15 min. Then, the supernatant was collected and the protein concentration was measured by BCA method. The protein samples in *in vitro* translation system were prepared by a special method. Once 50 μl translation reaction is completed, the 5 μl aliquot was removed, and it was added to 15 μl SDS sample buffer and heated at 75°C for 15 min to denature the proteins. The protein lysate (30 μg) was subjected to 10% SDS-PAGE and then electrotransferred onto the polyvinylidene difluoride membrane (PVDF, Millipore IPVH00010, Solarbio). And, the membrane was transferred to 5% skimmed milk for blocking for 1 h, followed by a membrane cutting process. Different bands were placed in an incubation box overnight in different primary antibodies. The main antibodies are as follows: β-actin (ab6276, Abcam), YRDC (sc-390477, SANTA CRUZ), and KRAS (12063-1-AP, Proteintech), and the ratios of them to the primary antibody dilution buffer (P0256, Beyotime) are 1:10,000, 1:800, and 1:5,000. The bands were washed the next day, and the secondary antibodies were incubated for 1 hour at room temperature. The ratio of β-actin and YRDC’s secondary antibody (SA00001-1, Proteintech) to the secondary antibody dilution buffer (P0258, Beyotime) is 1:10,000 and 1:5,000, and the ratio of KRAS’s secondary antibody (SA00001-2, Proteintech) to the secondary antibody dilution buffer (P0258, Beyotime) is 1:2,000. The bands were washed and soaked in the ECL kit (36208ES60, yeasen), and then ChemiDoc XRS + image analyzer (Bio-Rad, USA) was used to visualize the protein band, and Image Lab was used for immunoblot densitometric analysis. The experiment was repeated three times.

### Statistical Analysis

All data were described as mean ± standard deviation (mean ± SD). The comparison of two groups is carried out by Student’s t-test. All the other data were analyzed with one-way ANOVA followed by LSD when equal variances were assumed. The half maximal inhibitory concentration (IC_50_) was calculated by GraphPad Prism six software. All statistical analyses were performed by SPSS 22.0 or GraphPad Prism 6 (GraphPad Software, Inc. La Jolla, CA, United States). *p* < 0.05 was considered statistically significant.

## Results

### Effects of Lenvatinib on Cell Proliferation, Migration, and Clone Formation *In Vitro* Are Significantly Correlated With the Expression Level of YRDC

**FIGURE 1:**
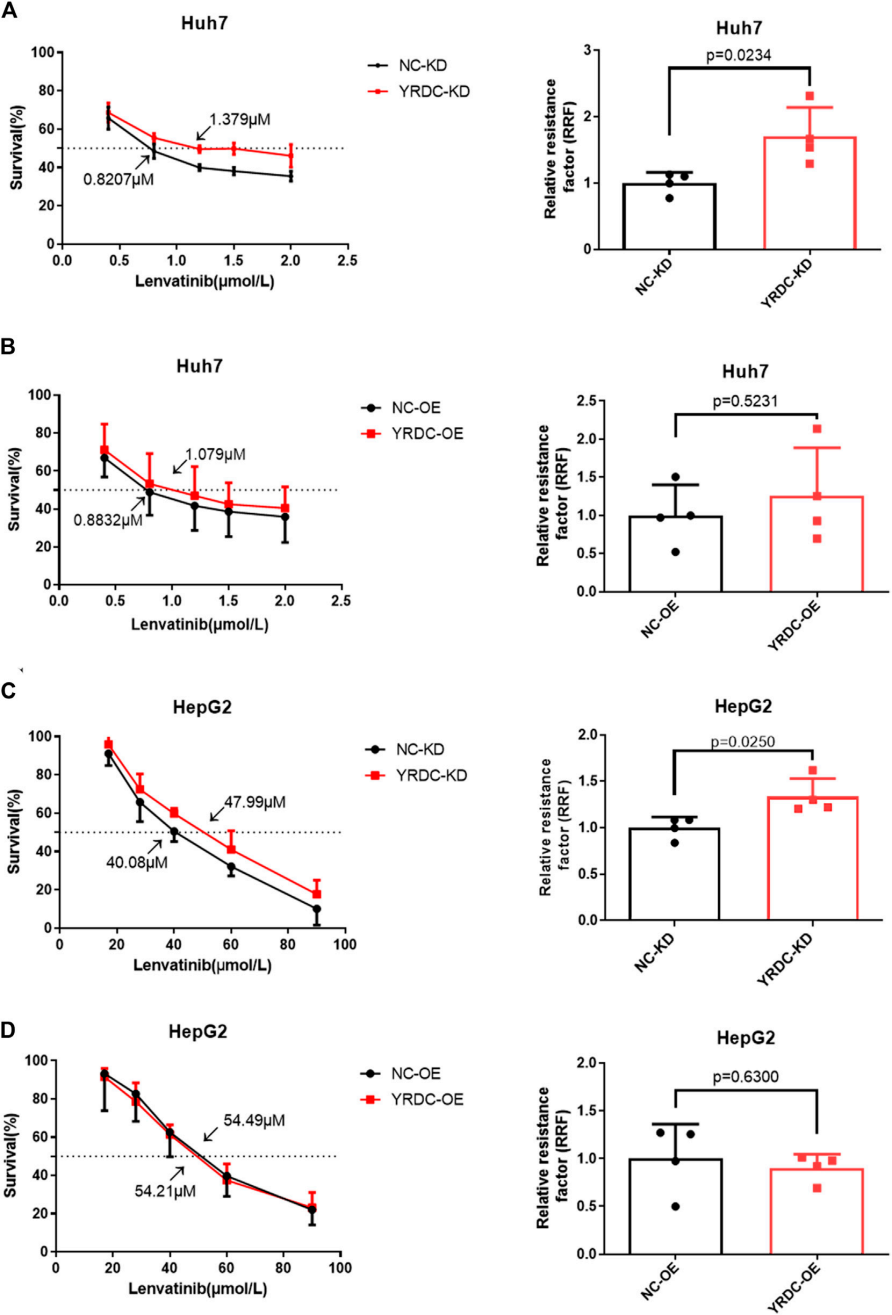
The knockdown of YRDC attenuates cellular sensitivity to lenvatinib in *in vitro* cell viability assay. **(A, B)** YRDC stable knockdown and overexpression in Huh7 cell were subjected to treatment with lenvatinib at various concentrations. The half maximal inhibitory concentration (IC_50_) was calculated by GraphPad Prism six software from four independent experiments. The relative resistance factor (RRF) of lenvatinib was calculated by dividing the IC_50_ of the control cells and by that of the cells with YRDC knockdown or overexpression. **(C, D)** YRDC stable knockdown and overexpression in HepG2 cell were subjected to treatment with lenvatinib at various concentrations. Dots in the graphs mean biological repetitions, *n* = 4.

**FIGURE 2:**
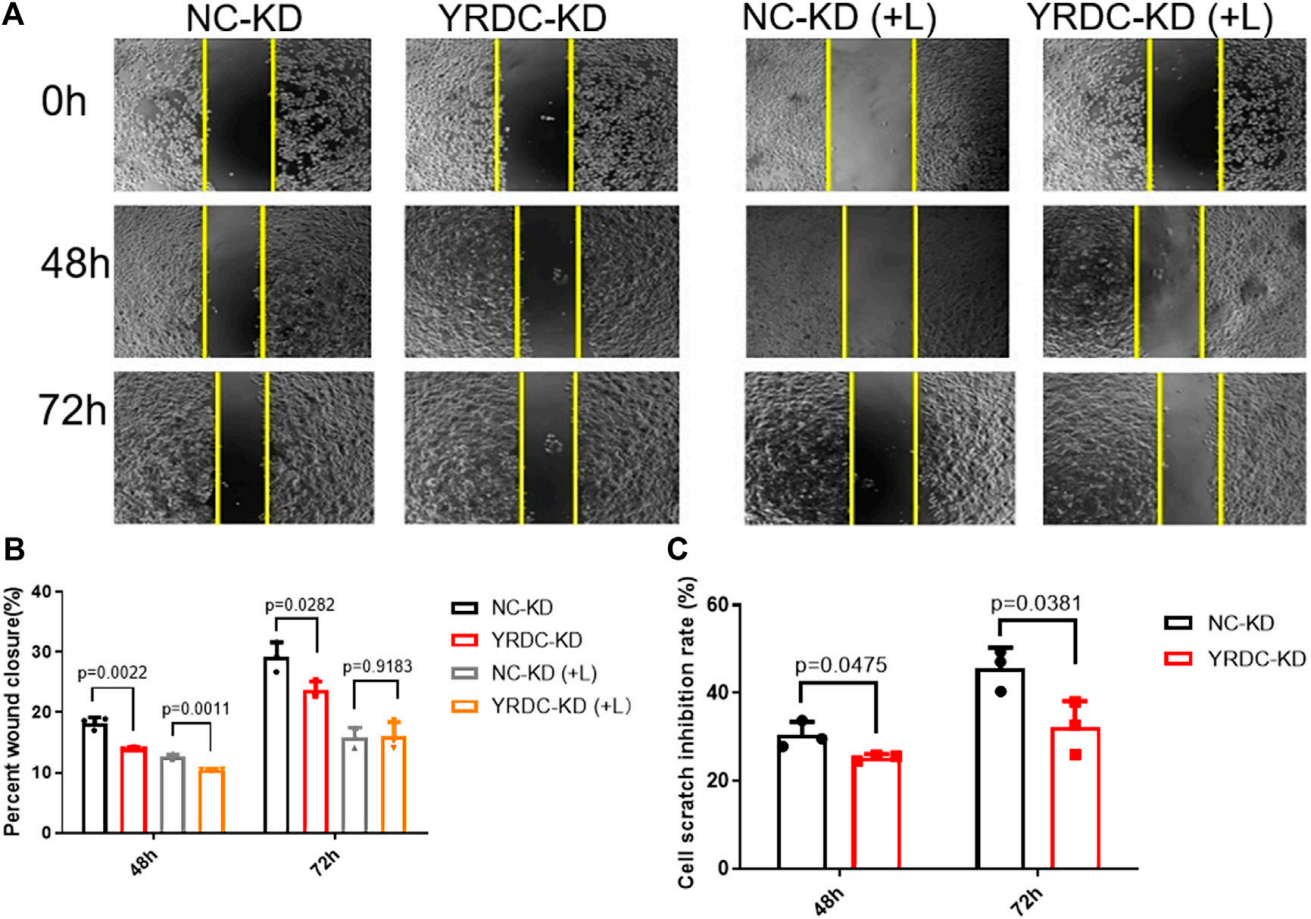
The knockdown of YRDC attenuates cellular sensitivity to lenvatinib in a wound-healing assay. **(A)** The knockdown of YRDC attenuates the antimigration effect of lenvatinib (0.8 μM) through the scratch experiment. **(B)** The migrating areas were measured at 48 and 72 h after the introduction of wounds in Huh7 cells with lenvatinib. **(C)** The cell scratch inhibition rate refers to the inhibition rate after lenvatinib addition. Dots in the graphs mean biological repetitions, *n* = 3.

**FIGURE 3:**
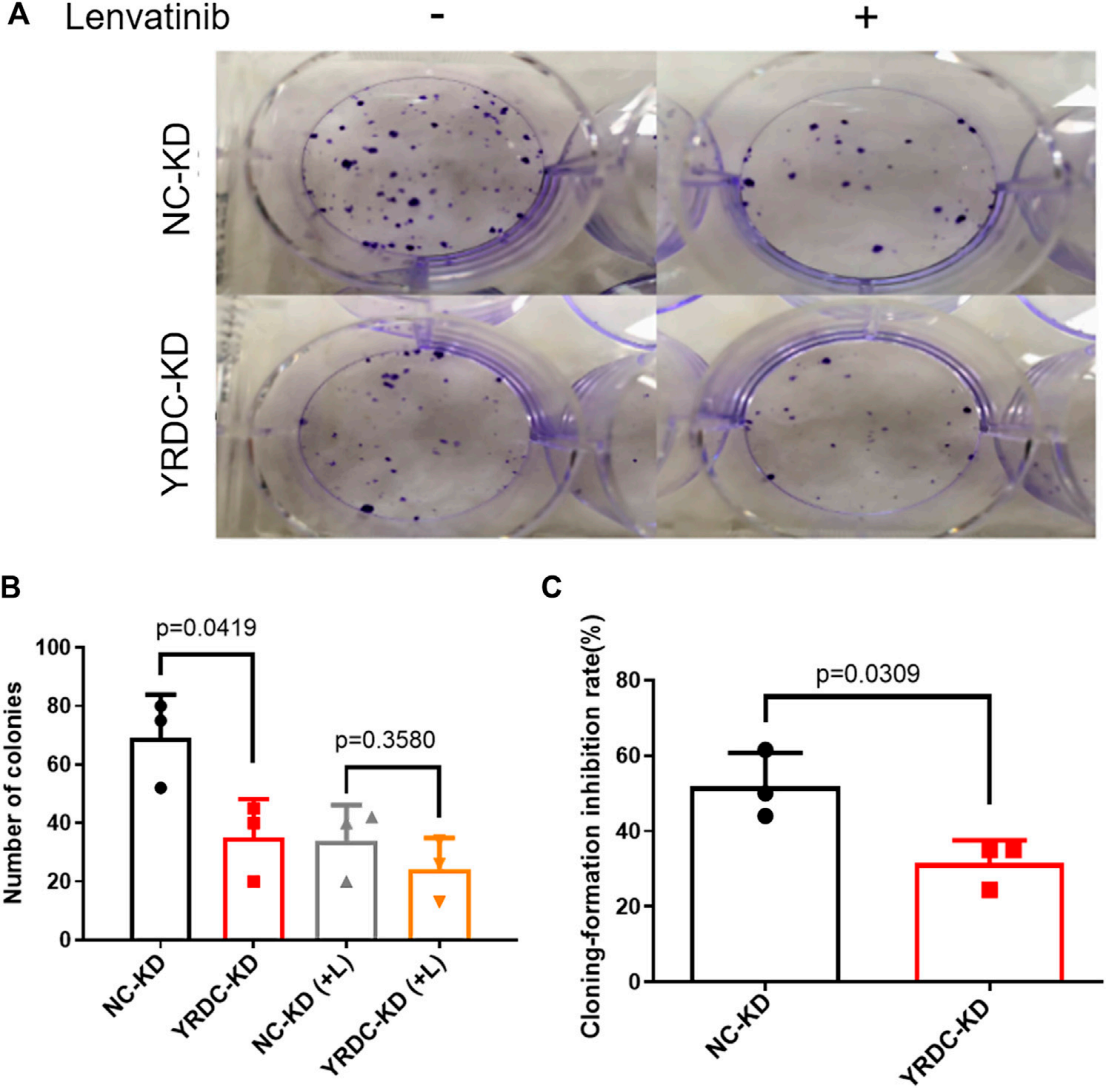
The knockdown of YRDC attenuates cellular sensitivity to lenvatinib in the clone formation assay. **(A)** The knockdown of YRDC attenuates the anti–colony-formation effect of lenvatinib (0.1 μM). **(B)** The clonal cell number was measured after 15 days in Huh7 cells with lenvatinib. **(C)** The cloning-formation inhibition rate refers to the inhibition rate after lenvatinib addition. Dots in the graphs mean biological repetitions, *n* = 3.

Stable YRDC knockdown (YRDC-KD), YRDC overexpression (YRDC–OE) and their respective control HCC cells (NC–KD or NC–OE), were successfully established in our previous study (Huang et al., 2019). The effects of lenvatinib on cell proliferation were measured by the CCK-8 cell viability assay in these four kinds of Huh7 or HepG2 cell models. Figure 1A showed that the Huh7 cells with YRDC knockdown were significantly less sensitive to lenvatinib-mediated growth inhibition than their control cells (IC_50_: 1.379 vs 0.821 μM). However, the Huh7 cells with YRDC overexpression were a little higher, but not significantly sensitive to lenvatinib than their control cells (Figure 1B). The HepG2 cells had the same pattern as the Huh7 cells, but the IC_50_ of lenvatinib to the former is higher than the latter (Figures 1C,D). Wound-healing assays revealed that the migration inhibition of lenvatinib to Huh7 cells was decreased by YRDC knockdown (Figures 2A–C). The clone formation assay further confirmed that Huh7 cells with YRDC knockdown had a less sensitivity to lenvatinib than their control cells (Figures 3A–C). These data suggested that the knockdown of YRDC in Huh7 or HepG2 cells allowed the cells to become more resistant to lenvatinib.

### Effects of Lenvatinib on Tumor Growth *In Vivo* Are Significantly Correlated With the Expression Level of YRDC

**FIGURE 4:**
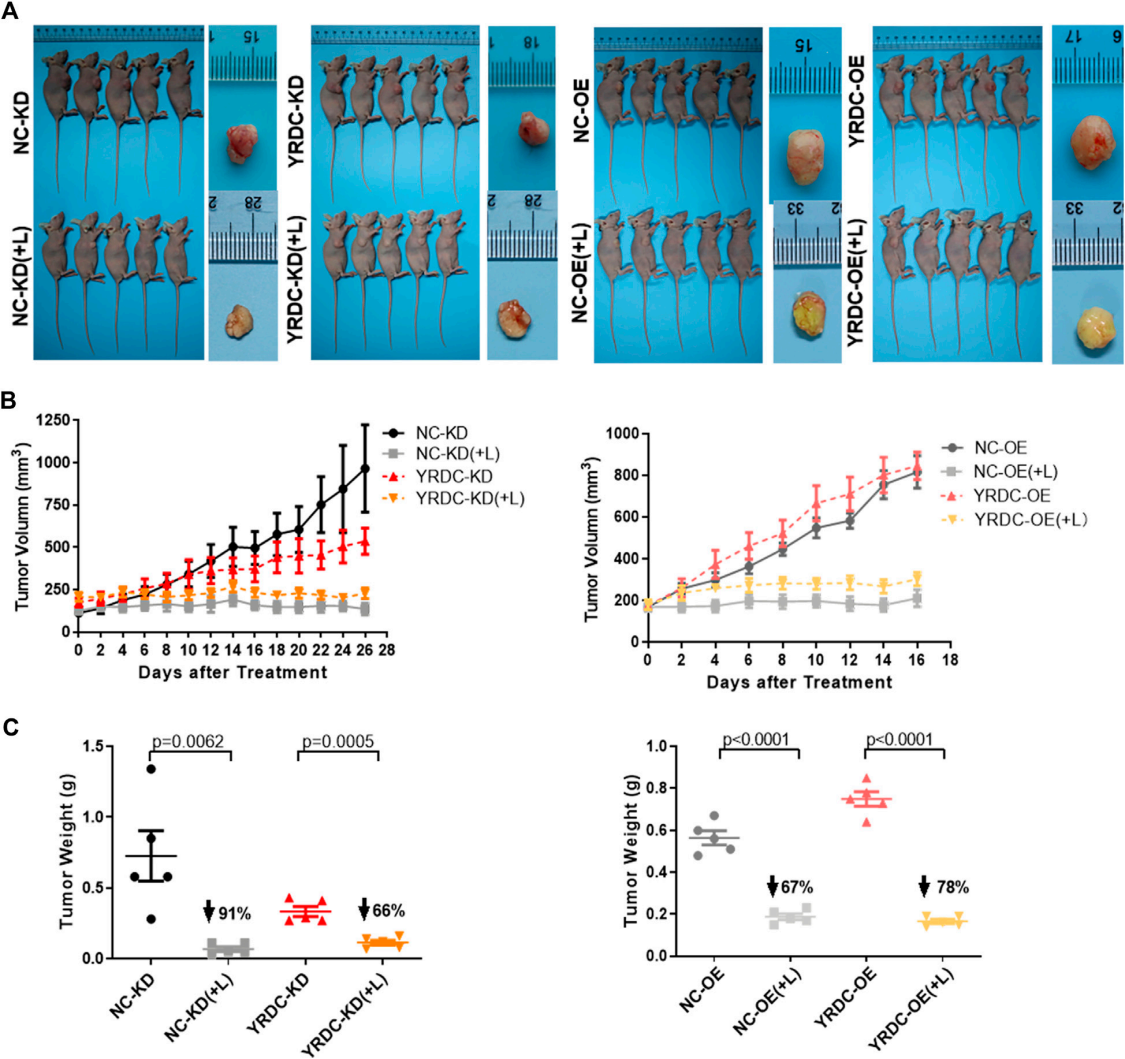
The effects of lenvatinib on tumor growth *in vivo* are significantly correlated with the expression level of YRDC. **(A)** The *in vivo* growth of xenograft tumors was significantly inhibited by lenvatinib at a dose of 30 mg/kg in all Huh7 cell models. **(B)** The tumor growth curves of YRDC knockdown or overexpression and their control cells after lenvatinib. **(C)** The difference of tumor weight with YRDC knockdown or overexpression cells after lenvatinib was compared with their controls. Dots in the graphs mean biological repetitions, *n* = 5.

To test the effect of YRDC on lenvatinib resistance in Huh7 cells *in vivo*, four kinds of stable Huh7 cells with different expression of YRDC were injected into nude mice for the construction of xenograft models. *In vivo* growth of xenograft tumors was significantly inhibited by lenvatinib at a dose of 30 mg/kg in all models (Figure 4A). The results indicated that YRDC knockdown or lenvatinib treatment significantly inhibited the tumor growth, exhibited as declined tumor volume (Figure 4B), and decreased tumor weight (Figure 4C). However, the knockdown of YRDC could weaken the lenvatinib-mediated tumor growth inhibition, mainly reflecting in the inhibition of tumor weight (66 vs. 91%, Figure 4C). Meanwhile, YRDC overexpression cells showed more sensitivity to lenvatinib compared with its control cells when evaluated by tumor weight (78 vs. 67%) (Figure 4C), however, not significantly different when evaluated by tumor volume (Figure 4B). These results suggested YRDC expression could affect the lenvatinib sensitivity on tumor growth *in vivo*, which mainly reflect in tumor weight.

### Lenvatinib Inhibits the Expression of YRDC in A Time-Dependent Manner

**FIGURE 5:**
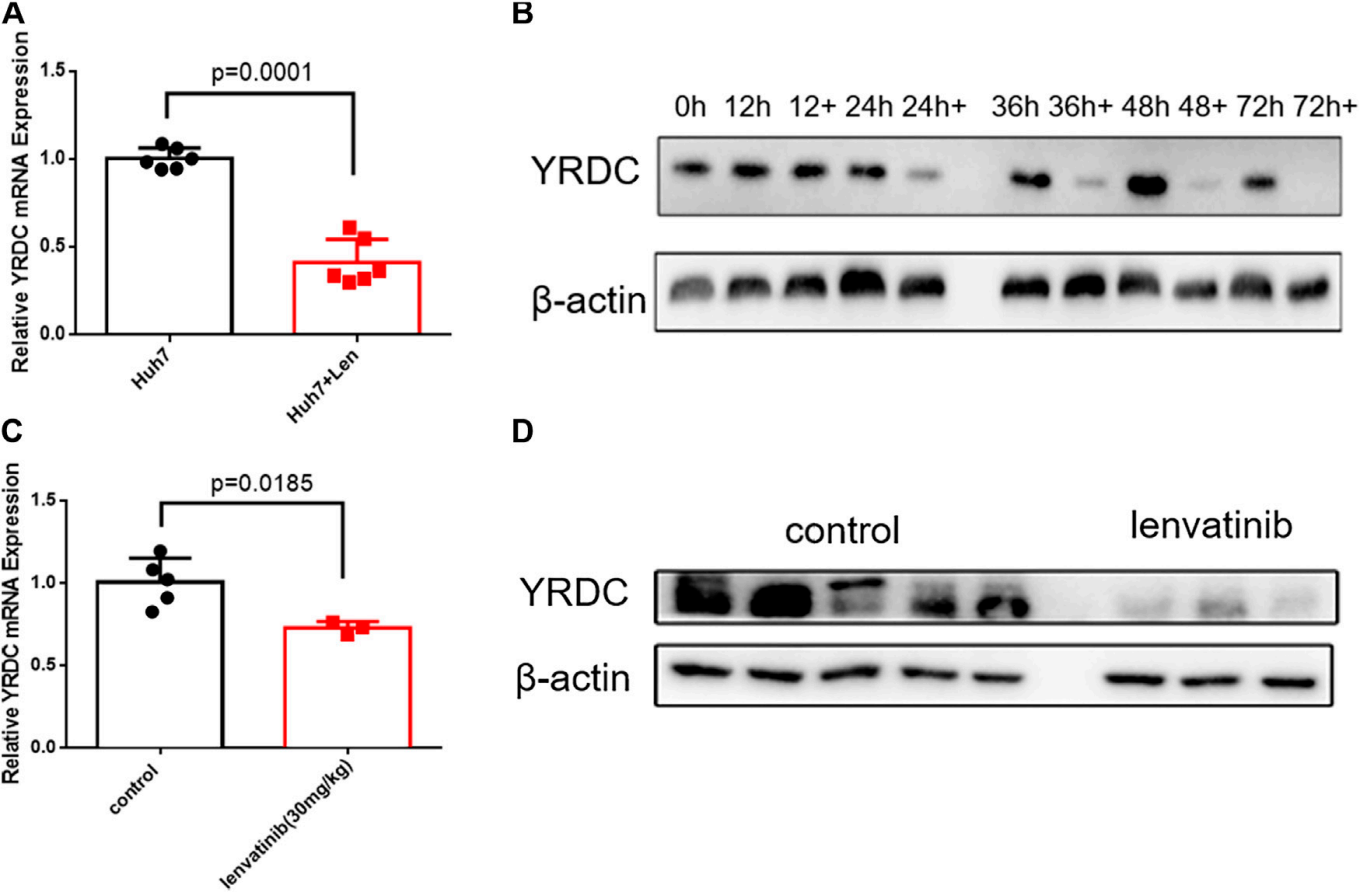
YRDC might be the target of anticancer of lenvatinib. **(A)** qPCR analysis for levels of YRDC in Huh7 cells with lenvatinib (0.8 μM) as well as the corresponding vehicle control, *n* = 6. **(B)** Lenvatinib (0.8 μM) inhibits the expression of YRDC in a time-dependent manner in Huh7 cells. **(C, D)** Lenvatinib inhibits the expression of YRDC in the xenograft of Huh7 cells in null mice, *n* = 5.

Since YRDC could be induced under stress, like the ischemia–reperfusion condition (Jiang et al., 2005), more evidence suggested that the resistance emerges soon after the initial treatment with lenvatinib (Fu et al., 2020). We wonder how YRDC changed under the treatment of lenvatinib. Interestingly, the mRNA and protein expression level of YRDC was remarkedly inhibited by lenvatinib in a time-dependent manner in Huh7 cells (Figures 5A,B). Subsequently, we further confirmed the inhibition effect of lenvatinib on YRDC expression in xenograft models (Figures 5C,D). Combined with the results above, we speculated that the inhibition effect of lenvatinib on YRDC could aggravate lenvatinib resistance in HCC cells with a low original expression of YRDC. Additionally, due to the inhibition of lenvatinib on YRDC, the expression of YRDC would be lower after lenvatinib treatment in original sensitive HCC cells (Figures 5A,B), and it may be an underlying cause due to which the resistance emerges soon after lenvatinib initial treatment. However, the detailed mechanisms of YRDC involved in lenvatinib resistance in HCC cells were still unknown.

### YRDC Regulates the Protein Translation of KRAS With a High Ratio of ANN Codons via Participating in the t^6^A Modification in NNU-tRNAs

**FIGURE 6:**
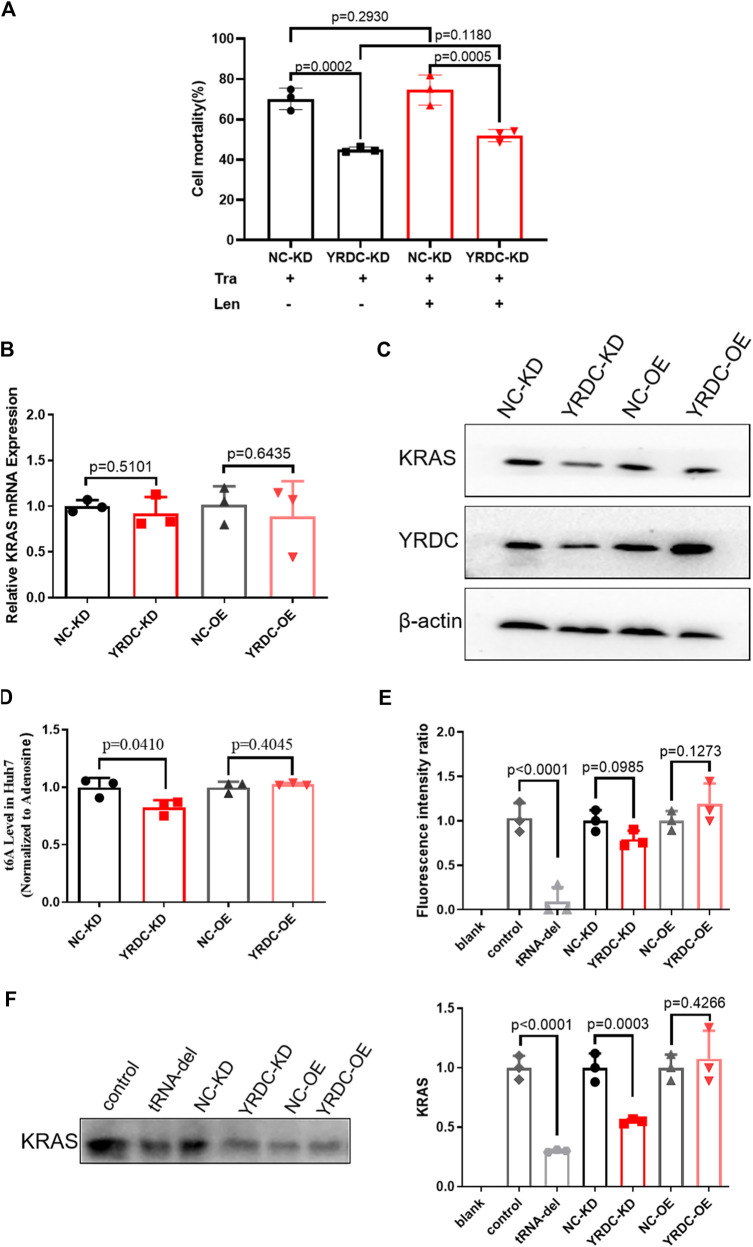
The anti-cancer effect of YRDC might affect the KRAS protein levels, but not mRNA levels. **(A)** The knockdown of YRDC attenuates cellular sensitivity to trametinib. After inhibiting the activity of the MAPK pathway, the expression of YRDC no longer affected the sensitivity of lenvatinib. **(B)** The mRNA expression levels of KRAS in overexpression, knockdown, and control in Huh7 cell. **(C)** The protein expression levels of YRDC and KRAS in overexpression, knockdown, and control in the Huh7 cells. **(D)** t^6^A levels in four kinds of Huh7 cells with different YRDC expression. **(E)** The fluorescent activity in addition of tRNAs from four kinds of Huh7 cells with different expressions *in vitro* reticulocytes translation system. **(F)** The KRAS level in addition of tRNAs from four kinds of Huh7 cells with different expressions *in vitro* reticulocytes translation system. Dots in the graphs mean biological repetitions, *n* = 3.

The results mentioned above suggested that YRDC mediated the resistance of lenvatinib in HCC cells. Our previous study reported that the expression level of YRDC was positively correlated with the proliferation, migration, and invasion of Huh7 cells. These suggested that YRDC could be the target and the executor of lenvatinib antitumor. As we know, the antitumor effect of lenvatinib was realized via inhibiting tumor angiogenesis and directly blocking the RAS/RAF/MEK/ERK signaling pathway. Considering our previous finding that YRDC promotes HCC cells proliferation via regulating the activity of the RAS/RAF/MEK/ERK pathway, we speculated that this pathway may involve in YRDC-mediated lenvatinib resistance. Further experiments were conducted to investigate the correlation between them. Trametinib, a selective MEK inhibitor, was used in our following *in vitro* experiments. It showed that knockdown of YRDC attenuates cellular sensitivity to trametinib (Figure 6A). When combined with lenvatinib treatment, the cell mortality did not decrease any further than trametinib treatment alone (Figure 6A). In other words, there was no difference in the sensitivity of lenvatinib in YRDC knockdown cells and its control cells after trametinib treatment. All these results confirmed that the RAS/RAF/MEK/ERK pathway was involved in the YRDC-mediated lenvatinib resistance.

YRDC was an NNU-tRNA t^6^A modification enzyme. The low t^6^A modification of NNU-tRNA reduced the translation rate or increased the possiblity of the translation error. We analyzed the codon composition of all genes related in the lenvatinib antitumor pathway and found that the KRAS genes have the highest ANN codon ratio (35.98%, Table 1). In the four kinds of Huh7 cell models, the mRNA expression of the KRAS had no difference (Figure 6B); however, the protein level of the KRAS in YRDC knockdown was significantly lower compared with its control (Figure 6C). The protein level of KRAS in YRDC overexpression cells was slightly higher compared with its control cells. These results suggested that the low expression of YRDC may mainly reduce the level of NNU-tRNA t^6^A modification and further decrease the protein synthesis of KRAS gene with a high ANN codon ratio.

**TABLE 1:** The codon composition of all genes related in the lenvatinib antitumor pathway.

Gene	Total codon count	ANN codon count	ANN codon ratio (%)	Rank	Gene	Total codon count	ANN codon count	ANN codon ratio (%)	Rank
KRAS	189	68	35.98	1	PI3K	1,660	438	26.39	37
BRCA1	1793	616	34.36	2	FasL	282	73	25.89	38
NRAS	190	62	32.63	3	FOX O 3	674	174	25.82	39
PTPRR	413	134	32.45	4	HRAS	190	49	25.79	40
PTEN	404	131	32.43	5	Grb2	218	56	25.69	41
RASGRF1	1,182	377	31.90	6	FGFR1	823	211	25.64	42
KIT	977	307	31.42	7	PDK1	557	142	25.49	43
sap1a	406	127	31.28	8	MYC	440	112	25.45	44
RAPGEF2	1,487	463	31.14	9	MYC	440	112	25.45	45
RAF1	649	200	30.82	10	MNK1	425	108	25.41	46
c-myb	450	138	30.67	11	EGF	1,153	291	25.24	47
GPCR	434	133	30.65	12	ERK1	380	95	25.00	48
MDM2	196	60	30.61	13	BCL-xl	234	58	24.79	49
RASGRF2	1,238	378	30.53	14	IRS1	1,243	308	24.78	50
RASGRF2	1,238	378	30.53	15	CCND1	296	73	24.66	51
MAPT	384	117	30.47	16	c-fos	381	93	24.41	52
CDK7	347	105	30.26	17	ARAF	610	148	24.26	53
PP2A	468	141	30.13	18	VEGFR3-s	1,299	313	24.10	54
PKC	707	213	30.13	19	VEGFR3-l	1,364	325	23.83	55
SRF	509	152	29.86	20	GSK3A	484	115	23.76	56
ERK2	361	107	29.64	21	DUSP1	368	86	23.37	57
MEK1	394	116	29.44	22	RASGRP2	610	139	22.79	58
RASGRP3	691	203	29.38	23	IKK	488	111	22.75	59
CDK9	373	109	29.22	24	CASP9	417	94	22.54	60
FGFR2	705	206	29.22	25	CREB	462	104	22.51	61
CDK8	465	135	29.03	26	HSP90AA1	733	248	33.83	62
EGFR	1,092	314	28.75	27	Elk1	429	92	21.45	63
VEGF	164	47	28.66	28	BAD	169	35	20.71	64
RASGRP	798	228	28.57	29	RASGRP4	674	137	20.33	65
RASGRP1	798	228	28.57	30	FGFR3	807	164	20.32	66
GSK3B	421	118	28.03	31	NOS3	1,204	242	20.10	67
CDK10	43	12	27.91	32	FGFR4	763	147	19.27	68
NFKB1	969	260	26.83	33	BCL2	240	46	19.17	69
MEK2	401	107	26.68	34	RET	459	84	18.30	70
TP53	215	57	26.51	35	p21	165	27	16.36	71
BRAF	767	203	26.47	36	p27	130	18	13.85	72

a The average level of ANN ratio in all genes is 26.16%.

Then we analyzed the t^6^A modification level in the four kinds of Huh7 cell models with different YRDC expressions. As expected, the t^6^A level in YRDC knockdown cells was significantly lower by 18% when compared with its control cells, and the t^6^A level in YRDC overexpression cells was not significantly changed when compared with its control cells (Figure 6D).

To further confirm this point, we established the *in vitro* rabbit reticulocyte translation system. Firstly, we eliminated the endogenous tRNA from the rabbit reticulocytes translation system and performed the *in vitro* translation of the luciferase gene under the addition of tRNA isolated from the four kinds of Huh7 cells. The fluorescent activity in tRNA from YRDC knockdown was slightly lower than that of its control (*p* = 0.0985). The situation reversed between YRDC overexpression and its control (Figure 6E). The ANN ratio in the luciferase gene was 28%, which is close to the average level 26.1% in all genes expressed in humans. This result indicated that YRDC knockdown could reduce general protein synthesis.

We further performed the *in vitro* translation of KRAS. The cDNA of KRAS was cloned into the pET-28a (+) plasmid. The KRAS mRNA was obtained by the reverse transcription using pET-28a (+)-KRAS plasmid as a template. In the *in vitro* rabbit reticulocyte translation system, the level of the KRAS protein was significantly lower when added with the tRNA isolated from YRDC knockdown cells compared with the tRNA from its control cells (0.55 vs. 1.0). However, there is no significance in the KRAS synthesis in addition of tRNA from YRDC overexpression cells compared with tRNA from its corresponding control cells (Figure 6F).

## Discussion

In the current study, we firstly found that the YRDC expression was associated with the sensitivity of lenvatinib in hepatocellular carcinoma cells. We also found that lenvatinib inhibited the expression of YRDC in a time-dependent manner. This effect may be an underlying cause due to which the resistance emerges soon after lenvatinib initial treatment. To investigate how YRDC modulates the sensitivity of lenvatinib, we assessed the effect of tRNA with different t^6^A levels on the translation of KRAS gene by the *in vitro* rabbit reticulocyte translation system and measured the expression levels of KRAS *in vitro* and *in vivo* experiments by western blot together with qPCR. We found that YRDC regulates the protein translation of KRAS in cells model, and the tRNA with low t^6^A modification level reduces the translation of KRAS in *in vitro* translation system. These results suggested that YRDC mediates the resistance of lenvatinib in HCC cells via modulating the translation of KRAS.

Based on the impressive finding from the REFLECT clinical trial, lenvatinib is now approved as the first-line therapy for patients with advanced HCC in Japan, the European Union, the United States, and China. However, the HCC patients with response to lenvatinib are still limited. There are almost half of HCC patients who develop resistance to lenvatinib in China (Fu et al., 2020). The understanding of how lenvatinib resistance happens seems to be more urgent in China. Our previous study found that YRDC promotes the proliferation of HCC cells via regulating the activity of the RAS/RAF/MEK/ERK pathway, which was the primary pathway of anticancer effect of lenvatinib. In our current study, we found that low YRDC expression significantly decreased the susceptibility of lenvatinib in Huh7/HepG2 cell models and in xenograft models. In addition, we found that lenvatinib remarkedly inhibited the expression of YRDC in a time-dependent manner. The inhibition effect of lenvatinib on YRDC could aggravate lenvatinib resistance in HCC cells. And, it may be an underlying cause of resistance emerges soon after lenvatinib initial treatment.

Receptors for growth factor, including VEGFR, PDGFR, and FGFR, were continuously activated in HCC and were critical to HCC development, progression, and metastasis. Lenvatinib has direct antitumor effects, as well as antiangiogenic properties, through inhibition of these receptors and their downstream cascade reactions, such as the RAS/RAF/MEK/ERK signaling pathway (Matsui et al., 2008a; Matsui et al., 2008b; Ikuta et al., 2009; Okamoto et al., 2013). Currently, we found that lenvatinib could inhibit the expression of YRDC in a time-dependent manner. Our previous study found that the expression of YRDC was dysregulated in the hepatocellular carcinoma tissues. The overall survival of patients with a high-level YRDC expression was shorter than that with low-level in patients. The low YRDC expression could significantly inhibit the activity of MEK/ERK and inhibit the proliferation of HCC cells *in vitro* as well as the tumor growth *in vivo*. All these results suggested that YRDC might be a target and an executor molecule of anticancer activity of lenvatinib. However, the detailed mechanisms of YRDC involved in lenvatinib resistance in HCC cells are still unknown. Exploring the detail mechanisms would provide the strategy for overcoming lenvatinib resistance in HCC and provide the biomarker for the prediction of lenvatinib outcome in HCC patients.

YRDC is involved in t^6^A biosynthesis of tRNA that recognizes ANN codons in *E. coli* and yeast. There are 16 kinds of these tRNA, including tRNA^Ile^_UAU_, tRNA^Ile^_GAU_, tRNA^Ile^_AAU_, tRNA^Lys^_UUU_, tRNA^Lys^_CUU_, tRNA^Asn^_GUU_, tRNA^Asn^_AUU_, tRNA^Arg^_UCU_, tRNA^Arg^_CCU_, tRNA^Ser^_GCU_, tRNA^Ser^_ACU_, tRNA^Thr^_UGU_, tRNA^Thr^_GGU_, tRNA^Thr^_CGU_, tRNA^Thr^_AGU_, and tRNA^Met^_CAU_ (Cantara et al., 2011; Kimura et al., 2014). We analyzed the ratio of these ANN codons corresponding to NNU-tRNA in all genes involved in the pathways of anticancer activity of lenvatinib. The ANN ratio of KRAS was ranked at top 1 (35.98%). The average ANN ratio in all genes was 26.16%. The protein content of KRAS in Huh7 cells with YRDC knockdown and *in vitro* translation system with tRNA isolated from YRDC knockdown was significantly lower compared with that in control cells. These results suggested that YRDC modulated the translation of key genes (KRAS) in pathways of anticancer activity of lenvatinib, which could be a partial reason that YRDC knockdown caused HCC cells resistance to lenvatinib. Other key genes with an enriched ANN codon, involved in the pathways of anticancer activity of lenvatinib, might be affected by a similar mechanism and should be explored in our future study. In the current study, we firstly provided the direct evidence on YRDC which modulated the translation of KRAS using the *in vitro* reticulocyte translation system. In our another study that is currently under the review, we also found that the translation of general protein in mouse with the liver-YRDC knockdown decreased, in particular, the proteins with a high ANN ratio decreased much more than those with a low ANN ratio.

Several multikinase inhibitors, including sorafenib, lenvatinib, sunitinib, ramucirumab, regorafenib, and cabozantinib, have recently been introduced into clinical practice for HCC (Abou-Alfa et al., 2018; Bruix et al., 2017; De Luca et al., 2020; Liu D. et al., 2018). In our current study, we give high priority to investigating sorafenib and lenvatinib, which are the first-line systemic therapies for HCC. Compared with sorafenib, lenvatinib has a distinctive feature, i.e., the potent activity against FGFR1-4. More evidence suggests that the activation of FGF signaling pathways in HCC contributes to its malignancy (Futami et al., 2017; Hagel et al., 2015; Miura et al., 2012; Paur et al., 2015; Sawey et al., 2011; Schmidt et al., 2016). The major downstream cascade reaction associated with FGFR in HCC is the RAS/RAF/MEK/ERK pathway (Li et al., 2016). In our study, the Huh7 cells with different expressions of YRDC only demonstrated the varied sensitivity to lenvatinib, but not to sorafenib (Supplementary Figure S1A, B). And, we also found that the Huh7 cells with different YRDC expressions showed the same response manner to pan-FGFR inhibitor BGJ (Supplementary Figure S1C). Furthermore, as mentioned above, the inhibition of lenvatinib on YRDC expression did not exist anymore after the treatment with the MEK inhibitor (Figure 6A). It suggested that the FGFR-related RAS/RAF/MEK/ERK pathway was mainly involved in YRDC-mediated lenvatinib resistance. This result might also be related to a recent finding that the expression level of FGF19 was correlated with lenvatinib susceptibility in HCC cells. In their study, the researchers found that FGF19 was downregulated in lenvatinib-resistant HCC cell lines, and the FGFR pathway plays a critical role in lenvatinib resistance (Myojin et al., 2021). However, whether there is a connection between YRDC and FGF19-mediated lenvatinib resistance is unknown. We could investigate that in the future study.

In addition to the mechanisms mentioned above, there are few other reported mechanisms of lenvatinib resistance in HCC. Lenvatinib-resistant HCC cells, established by culturing with long-term exposure to lenvatinib, were commonly used cell models in these studies. *In vitro* results showed that the vascular endothelial growth factor (VEGF), VEGFR2, platelet-derived growth factor-AA (PDGF-AA), and angiogenin were increased significantly in lenvatinib-resistant cells (Ao et al., 2021; Zhao et al., 2021). Both activation of the MAPK/MEK/ERK signaling pathway and the upregulation of epithelial mesenchymal transition (EMT) markers were observed in lenvatinib-resistant cells (Ao et al., 2021; Zhao et al., 2021). However, the researchers did not explore the detailed mechanism in these studies. The interferon regulatory factor 2 (IRF2) is a constitutive transcription factor associated with the development of various cancers by regulating the cancer cell growth, apoptosis, and drug resistance. A recent study found that IRF2 promoted proliferation, inhibited apoptosis, and increased lenvatinib resistance of HCC cells by regulating *β*-catenin expression (Guo et al., 2021). In brief, the mechanisms underlying lenvatinib resistance are still very limited and are worth exploring.

In conclusion, lenvatinib is a promising option in anticancer treatment for advanced HCC. However, lenvatinib resistance limits the clinical use in HCC. YRDC expression was positively correlated with the sensitivity of HCC to lenvatinib in cell models and *in vivo* xenograft model. The detail of the mechanism is that YRDC mediates the resistance of lenvatinib in hepatocarcinoma cells via modulating the translation of KRAS. The mRNA expression and protein expression level of YRDC in tissue-biopsy would be measured by qPCR and IHC-P in clinical. In future study, we would further investigate whether the expression of YRDC or functional SNP of *YRDC* might be regarded as a biomarker for the prediction of lenvatinib efficacy in clinical practice.

## Data Availability

The original contributions presented in the study are included in the article/Supplementary Material. Further inquiries can be directed to the corresponding authors.
