# Supplementary figures and images for: YRDC Mediates the Resistance of Lenvatinib in Hepatocarcinoma Cells via Modulating the Translation of KRAS
Source: Front Pharmacol. 2021 Oct 1;12:744578. doi: 10.3389/fphar.2021.744578 (PMC8517968; doi:10.3389/fphar.2021.744578)

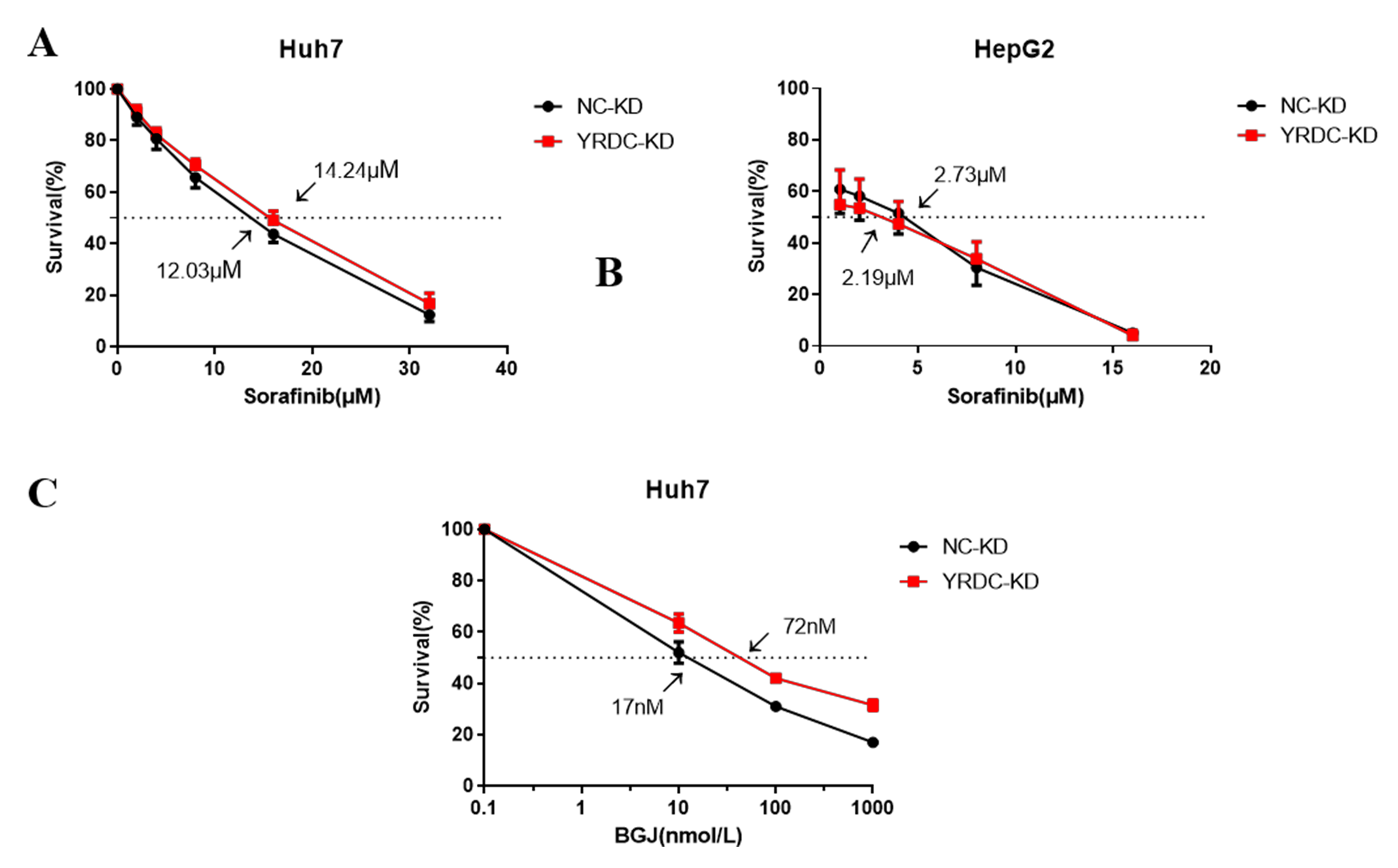

Supplement: Supplementary file 1 [file Image1.TIF]
